# Rediscovery of Chamisso’s type specimens of Hawaiian *Psychotria* (Rubiaceae, Psychotrieae) in the herbarium of the Natural History Museum, Vienna

**DOI:** 10.3897/phytokeys.114.29426

**Published:** 2018-12-20

**Authors:** Andreas Berger

**Affiliations:** 1 Department of Botany and Biodiversity Research, University of Vienna, Rennweg 14, A-1030, Vienna, Austria University of Vienna Vienna Austria

**Keywords:** Lectotypification, *
Psychotria
*, Hawaii, Chamisso, herbarium history

## Abstract

Between 1815 and 1818, Count Nikolai Romanzoff funded an expedition of the Russian brig *Rurik*. Besides their primary goal to discover the Northeast Passage, their aim was to collect scientific specimens, for which the botanist Adelbert von Chamisso and the entomologist Johann Friedrich von Eschscholtz were commissioned. On the Hawaiian Islands, they collected two unknown endemic species that Chamisso and Diederich Franz Leonhard von Schlechtendal later described as *Coffeakaduana* and *C.mariniana*, both now assigned to the large and complex genus *Psychotria* (Rubiaceae, Psychotrieae). The private herbarium of Chamisso is now preserved at the Komarov Botanical Institute, St. Petersburg (LE). In the late 1930s, their type collections of *Psychotriakaduana* and *P.mariniana* were sent out on loan for study, but got lost in transit during the aftermath of the Second World War. No extant original material was found during a subsequent revision of Hawaiian *Psychotria* and both species were consequently neotypified. These neotypes are superseded by the here-reported rediscovery of original material in the herbarium of Stephan Ladislaus Endlicher preserved at the Natural History Museum, Vienna (W) and these specimens are here designated as lectotypes. As both are rather fragmentary, the former neotypes are additionally designated as epitypes. In addition, some peculiarities and details of the expedition and its collections are noted.

## Introduction

### The Romanzoffian expedition, Chamisso and his collections

Between August 1815 and August 1818, Count Nikolai Romanzoff (1754–1826), Chancellor of the Russian Empire and a patron of science, commissioned an expedition around the world on the Russian brig *Rurik* under the command of captain and cartographer Otto von Kotzebue (1787–1846). Besides their primary goal to find the Northeast Passage from the Bering Strait to the Atlantic Ocean, their aim was to collect scientific specimens of all kinds, for which the botanist and famous poet Ludolf Karl Adelbert von Chamisso (1781–1838) and the zoologist Johann Friedrich von Eschscholtz (1793–1831), as well as the artist Louis Choris (1795–1828), were hired. A detailed description of Chamisso’s life, works and the Romanzoffian Expedition was given by Bździach (2004) and Maaß (2016).

Whilst the expedition was not able to realise their nautical goal, they brought together ample collections of plants, animals and other objects, largely from the Pacific region. In his first report to Romanzoff, Chamisso (1818: 206) estimated that they had collected around 2,500 species of plants with a third of them being undescribed. To date, the total extent of their collections, including duplicates, remains unknown. After returning to Europe, Chamisso was allowed to take his botanical collections to Berlin for study and publication and the bulk of them remained there until his death (Hiepko 2004; Maaß 2016: 134; see also Chamisso 1818: 208).

In June 1819, Chamisso became an adjunct (“Zweiter Kustos”) in the Berlin Botanical Garden, at that time located in Schöneberg. Amongst other tasks, he was commissioned with creating a garden herbarium, a duty he increasingly neglected in favour of working on his collections from the Romanzoffian expedition (Urban 1917: 16, 19; Schmid 1942; Hiepko 2004). In 1833 Chamisso succeeded his friend Diederich Franz Leonhard von Schlechtendal (1794–1866) as curator of the Royal Herbarium Berlin (B), a position he kept until his death (Schlechtendal 1839; Urban 1917: 20). Together they published ten volumes of the “De plantis in expeditione speculatoria Romanzoffiana observatis” (e.g. Chamisso and Schlechtendal 1826a, 1826b, 1827, 1829a, 1829b).

In that series, they described ca. 60 new genera and 1,150 species (http://www.ipni.org; retrieved June 2018). A large portion (ca. 50 gen. and 700 spp.) of these names was based on material collected during the expedition, but other specimens such as Brazilian collections by Friedrich Sellow were also included (Imchanitzkaja 2004: 124; Maaß 2016: 139). Chamisso and Schlechtendal worked on the bulk of the collections themselves. However, certain families were assigned to specialists such as Georg Friedrich Kaulfuß (ferns), Christian Friedrich Lessing (Asteraceae) and Carl Bernhard Trinius (Poaceae), in part explaining the dissemination of many of Chamisso’s specimens (e.g. Schlechtendal 1839; Lasègue 1845; Schmid 1942: 10; Maaß 2016: 171–172).

Chamisso presented a complete set of specimens from the expedition to the Berlin Herbarium. Unfortunately, these specimens were destroyed during a bomb raid that hit the herbarium during the Second World War (Hiepko 1987). As specified in his will, an additional set of “1,800 plant species” was presented to his successor at B, Johann Friedrich Klotzsch, who generously donated them to the herbarium (Schlechtendal 1839: 104; Urban 1917: 19, 22, 336) and these were likewise destroyed. His colleague Schlechtendal, professor of botany and director of the Botanical Garden of the University Halle (HAL), also received duplicates for his extensive private herbarium, which was later purchased by HAL and is still extant (Werner 1988; Braun and Wittig 2003: 14).

In 1840, two years after Chamisso’s death, his private herbarium containing 10,000 to 12,000 species (Ruprecht 1864: 4) and ca. 60,000 specimens, was purchased by the Botanical Museum of the St. Petersburg Academy of Sciences. This collection with the specimens from the Romanzoffian expedition and other regions, as well as collections from ca. 60 other botanists, is now at the Komarov Botanical Institute in St. Petersburg (LE), where it is kept separately. Several additional duplicates of some of Chamisso’s collections came to LE via the purchase of other private herbaria. Likewise, LE acquired the herbarium of his fellow member of the Romanzoffian expedition, Eschscholtz, in 1825 (Urban 1917: 336; Imchanitzkaja 2004; Maaß 2016: 145, 171).

### Taxonomic history of Hawaiian *Psychotria* L.

The genus *Psychotria* (Rubiaceae, Psychotrieae) is a speciose pantropical group, comprising mainly understorey shrubs from wet forests. Due to the large number of morphologically similar species and long-unclear generic boundaries with respect to related genera, *Psychotria* was long perceived as a taxonomic nightmare (e.g. Sohmer 1977: 103). However, DNA-phylogenetic studies and a re-interpretation of morphological characters have recently improved our understanding of the group (e.g. Nepokroeff et al. 1999; Razafimandimbison et al. 2014, 2017). These studies have led to a narrower circumscription of the tribe Psychotrieae now including the single genus *Psychotria* and the transfer of all related genera such as *Palicourea* Aubl. to a separate tribe, Palicoureeae. In addition, many species of *Psychotria* have been transferred to *Palicourea*, once thought to contain only species with conspicuous flowers adapted to hummingbird-pollination (e.g. Taylor et al. 2010; Taylor 2015a, 2015b, 2017, 2018; Taylor and Hollowell 2016; Berger 2017, 2018).

As currently circumscribed, the genus *Psychotria*, as well as the tribe Psychotrieae, has its centre of diversity in the Paleotropics and harbours at least 1,600 species. Within the Rubiaceae, the group is largely diagnosed by the presence of raphides, valvate corolla aestivation and the frequent occurrence of heterostyly (subfamily Rubioideae), as well as by a predominantly woody habit, mostly terminal inflorescences, single ovules per locule and predominantly fleshy and drupaceous fruits (Psychotrieae alliance). Within the alliance, a grey or reddish-brown drying colour, caducous stipules, inconspicuous whitish flowers, pyrenes without preformed germination slits and seeds with an alcohol-soluble red seed-coat pigment generally characterise the Psychotrieae and the genus *Psychotria*. The opposite character states are variously found in *Palicourea*, as well as in other Palicoureeae (e.g. Taylor 1996; Razafimandimbison et al. 2014). In addition, species of the Palicoureeae are phytochemically differentiated from the Psychotrieae by largely accumulating alkaloids (e.g. Berger et al. 2012, 2015, 2016, 2017; Schinnerl et al. 2012), cyclotides (Koehbach et al. 2013) and different groups of flavonoids (e.g. Berger et al. 2016).

*Psychotria* is the only genus of the Psychotrieae and Palicoureeae that reached the Hawaiian Islands, where it forms a characteristic component of the native mesic to wet rain forests (Sohmer 1977, 1978; Wagner et al. 1999; Nepokroeff et al. 2003). The *Rurik* visited the islands in 1816 as well as in 1817. Whilst they did not stay on the island of Hawaii (“Big Island”) for more than a “quick touch on the beach”(Chamisso 1826: 7, 1836a: 207, 210–211), they dropped anchor at Honolulu, at that time the largest port on the Hawaiian Islands and stayed there for a total of roughly four weeks. According to Chamisso’s itinerary, they largely collected in the valleys around Honolulu. During two excursions that lasted several days, they also explored the two major mountain ranges and reached elevations of up to 730 m (Chamisso 1826: 7–8, 1836a).

Based on the specimens collected on Oahu, Chamisso and Schlechtendal described the first two Hawaiian species of *Psychotria* under the names *Coffeakaduana* Cham. & Schltdl. and *C.mariniana* Cham. & Schltdl. (Chamisso and Schlechtendal 1829a). Asa Gray (1858) subsequently transferred both to the newly established genus *Straussia* (DC) A.Gray and also added a new species (*S.hawaiiensis* A.Gray). Subsequently, numerous authors published new species and intraspecific taxa for both *Psychotria* and *Straussia*, thus considerably raising the number of Hawaiian taxa. Later, Francis Raymond Fosberg (1964) synonymised *Straussia* with *Psychotria* and provided the necessary combinations such as for the two above-mentioned species currently known as *P.kaduana* (Cham. & Schltdl.) Fosberg and *P.mariniana* (Cham. & Schltdl.) Fosberg.

More recently, Seymour Sohmer (1977) published an extensive review of Hawaiian *Psychotria*, reduced many names to synonymy and recognized 11 endemic species with 8 varieties within two endemic sections. As currently understood, these species form a monophyletic group and are the result of a single introduction to the Hawaiian Islands, with subsequent colonisation from the oldest to the youngest islands followed by radiation and speciation events partly accompanied by polyploidisation (Kiehn 1996; Nepokroeff et al. 2003; Kiehn and Lorence in review).

### Chamisso’s collections of Hawaiian *Psychotria*

The Romanzoffian Expedition was amongst the first scientific expeditions that touched the Hawaiian Islands and studied their native flora. As mentioned above, they collected the first specimens of the species currently known as *Psychotriakaduana* and *P.mariniana*. Type specimens of both should be expected at LE and several other herbaria (see above). However, no such specimens were catalogued in the LE digital herbarium (https://www.binran.ru/collections; retrieved June 2018) or the JSTOR Global Plants database (http://plants.jstor.org; retrieved June 2018). Likewise, no specimens are extant in the Berlin Herbarium and none has been found in the private herbarium of Schlechtendal at HAL (Braun and Wittig 2003). This appears to confirm the opinion of Sohmer (1977), who could not trace any original material in his extensive revision of Hawaiian *Psychotria*.

As part of his studies on various groups of Pacific Rubiaceae, Fosberg was the last taxonomist to see Chamisso’s type collections of *P.kaduana* and *P.mariniana* at LE (Fosberg 1964). He received these specimens on loan shortly before the outbreak of the Second World War. After the war, he returned them to the Russian Embassy for return to LE. Unfortunately, though, the specimens never arrived or resurfaced elsewhere and were probably lost in the aftermath of the war. As no other original material could be located, Sohmer (1977) designated a neotype for each of these names (ICN, Melbourne Code, McNeill et al. 2012, Art. 9.7) and expressed his hope that they “will be supplanted eventually by the recovery of the holotype”.

Although the present study cannot satisfy Sohmer’s hopes for a rediscovery of the lost types from LE, the discovery of duplicates of these in the collection of the herbarium of the Natural History Museum (W) is reported here. These specimens are original material, so they supersede the neotypes (ICN, Art. 9.19) and are designated here as lectotypes. In order to maintain nomenclatural stability, the neotypes of Sohmer (1977) are additionally designated as epitypes to support these rather fragmentary lectotypes (ICN, Art. 9.8).

## Taxonomic treatment

### Psychotria kaduana

Taxon classificationPlantaeGentianalesRubiaceae

(Cham. & Schltdl.) Fosberg, Occas. Pap. Bernice Pauahi Bishop Mus. 23(2): 43, 1962.

Coffea kaduana Cham. & Schltdl., Linnaea 4(1): 33–35, 1829a. ≡ Straussiakaduana (Cham. & Schltdl.) A.Gray, Proc. Amer. Acad. Arts 4: 43, 1860. **Type.** USA. Hawaii: Oahu, Southern Waianae Range, < 730 m alt., 7–10 Oct 1817, or, Southern Koolau Range, < 730 m alt., 12 Oct 1817, *L.K.A. von Chamisso s.n.* (lectotype, designated here: W-Endl. 0065914!); Oahu, Kahuauli Ridge, 500–750 m alt., 17 Dec 1931, *E. Christophersen & E. Hume 1426* (epitype, designated here: BISH barcode 1010994!, Sohmer 1977: fig. 52!).

#### General remarks.

*Psychotriakaduana* (sect.Straussia (DC.) Fosberg) is the most widely distributed species of Hawaiian *Psychotria*. It is found on the islands of Kauai, Oahu, Molokai, Lanai and Maui. It possesses a wide ecological amplitude resulting in considerable morphological variation and many local forms, some of which have received taxonomic recognition. However, it was shown that these all intergrade and are best treated as a single polymorphic species. A detailed synonymy and description of *P.kaduana* including lists of specimens, distribution maps, drawings and photos illustrating morphological variations are found in Sohmer (1977: 148–159) and Wagner et al. (1999). According to molecular phylogenetic data, this species belongs to the “*greenwelliae*” clade, which also comprises *P.fauriei* (H.Lév.) Fosberg, *P.greenwelliae* Fosberg, *P.hathewayi* Fosberg, P.hawaiiensisvar.hillebrandii (Rock) Fosberg and *P.mauiensis* Fosberg (Nepokroeff et al. 2003).

#### Etymology.

The protologue of *Psychotriakaduana* lacks information about the etymology of the name, but a person called “Kadu” is frequently mentioned in Chamisso’s publications. While visiting the Aur Atoll (Ratak Chain, Marshall Islands) in February 1817, the *Rurik* was approached by Kadu, a native of Woleai (“Ulea”), an atoll in the eastern Caroline Islands, Federated States of Micronesia. Four years before, a storm bore Kadu’s boat far to the east and, after months at sea, he finally reached the Ratak Chain. With curiosity and the intention of being dropped off closer to home, he joined the expedition and became a close friend of Chamisso and his prime source of ethnographic information on Micronesia. After visiting distant places such as Alaska and Hawaii, the expedition returned to the Ratak Chain in November 1817, where Kadu finally settled in the Wotje Atoll (Chamisso 1818: 203, 1836a: 278–280; Kotzebue 1821: 86–93; Maaß 2016; Igler 2017).

Chamisso wrote with great admiration about his “companion, teacher and dearest friend” Kadu, and dedicated to him the genus *Kadua* Cham. & Schltdl., a group of Pacific Rubiaceae-Spermacoceae with its centre of diversity on the Hawaiian Islands (Chamisso and Schlechtendal 1829b: 157–158 “Nomen in honorem amicissimi Kadu ex Ulea, dulcissimi nobis in expecitione Romanzoffiana per aliquot menses sodalist atque magistri”, Terrell et al. 2005). A naturalistic lithographed portrait of Kadu in his traditional dress is given by the expedition’s artist Choris (1822: Iles Sandwich, pl. 17). The same portrait with European dress is found in the honorary frontispiece of Chamisso’s expedition report (1836b), once more illustrating Chamisso’s connection with Kadu. Ultimately, it may not be clarified if the epitheton “kaduana” refers to a superficial resemblance with the genus *Kadua*, the person Kadu or both.

#### Typification.

The protologue of *Psychotriakaduana* gives the type information as “In nemorosis montium Insulae O-Wahu A. D. 1817 legimus” (Chamisso and Schlechtendal 1829a: 33–35). Information provided in the itinerary (Chamisso 1826: 7–8) allows dating of their second visit to Oahu and the period in which the type collection was made to the time between 2 and 14 October 1817. The diary of Chamisso (1836a: 344) provides additional information about their collecting activities and reports only two occasions where plants have been collected during that visit.

Between 7 and 10 October 1817, Chamisso explored the “western mountain range of the island” (Chamisso 1836a: 344), which he and his two guides climbed from around Pearl River. Likely, the information denotes the Waianae Range, the western of the two mountain ranges on the island of Oahu. During that time, Eschscholtz suffered from a sore leg, could not attend the expedition and was left in care of drying previous collections on board the *Rurik*. On the 12^th^ of October 1817, Eschscholtz had recovered and both made a daytrip to “the mountains”, this time denoting the Koolau Range behind Honolulu (Chamisso 1836a: 342, 344–347).

**Figure 1.**
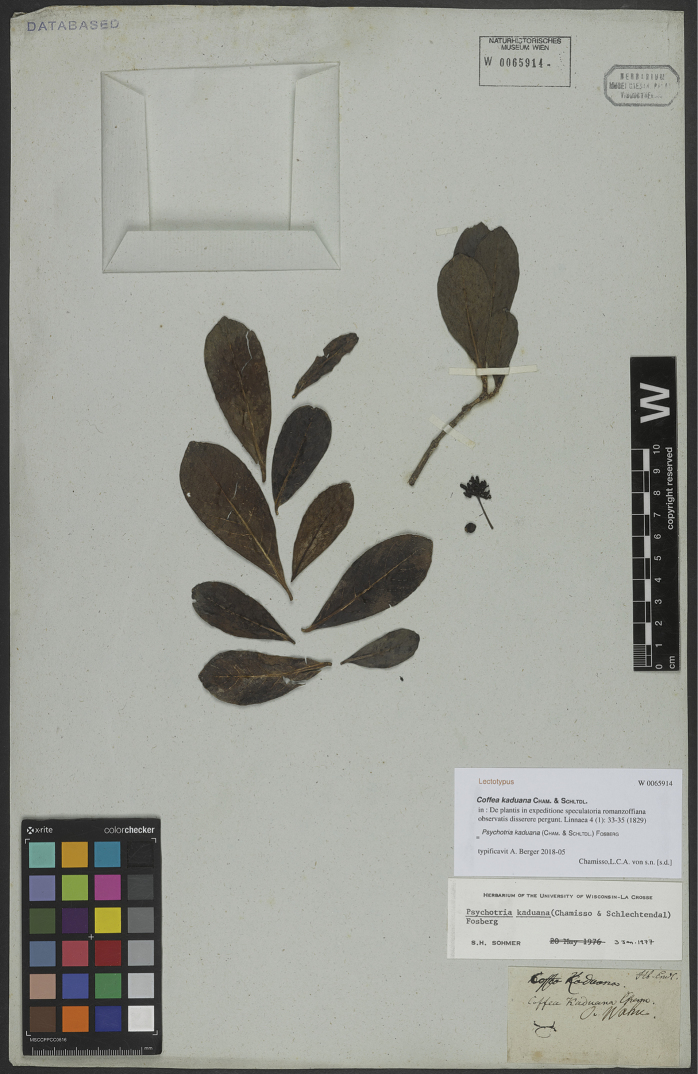
Lectotype of *Psychotriakaduana* (Cham. & Schltdl.) Fosberg collected by L.K.A. von Chamisso during the Romanzoffian Expedition in 1817 (*L.K.A. von Chamisso s.n.*, W-Endl. 0065914). The sheet originates from the private herbarium of S.L. Endlicher, now preserved at the Herbarium of the Natural History Museum, Vienna. Photo: Courtesy of the Natural History Museum, Vienna.

At the Herbarium of the National History Museum (W), a peculiar specimen of *P.kaduana* is preserved (Figure 1). The sheet contains a small sterile branchlet and a capsule with some leaves, an inflorescence and an immature fruit. The label gives the names “*Coffeakaduana*” and “*Coffeakaduana* Cham.” in two different hands as well as the island “O. Wahu”, but no information about the collector. On the upper right corner, “Hb. Endl.” in ink indicates that the specimen originated from the private herbarium of Stephan Ladislaus Endlicher (1804–1849) and which he presented to the Botanical Museum after he was appointed curator (Torrey 1836; Anonymous 1845). Sohmer (1977: 157) subsequently confirmed the identification of the specimen and cited it as “Oahu without specific locality, *Endlicher s.n.* (W)”.

Endlicher, however, never visited the Hawaiian Islands, so the respective specimen was not collected by him. The age of the specimen, the name and the locality on the label indicate that it could be original material of *Psychotriakaduana*. Comparison with specimens at LE (e.g. Imchanitzkaja 2004; Popov 2014; Maaß 2016: 149–170) and HAL (international herbarium database JACQ, http://herbarium.univie.ac.at/database) shows that Chamisso’s labels are characteristic in size, paper and handwriting and confirm that this specimen was indeed collected by Chamisso. Therefore, it constitutes type material for *P.kaduana*.

Concerning the history of the specimen, Endlicher was professor of botany, director of the Botanical Garden and the Botanical Museum of Vienna from 1839–1849 (Anonymous 1849). As one of the foremost systematicists, taxonomists and prolific writers of his time (e.g. Flora brasiliensis, Nova genera et species plantarum, Genera plantarum), he was in contact with many contemporary botanists. A letter preserved in the Berlin State Library (Endlicher 1833), shows that he was in correspondence with Chamisso and that they exchanged specimens. This could explain how Chamisso’s specimens from the Romanzoffian Expedition came into the possession of Endlicher.

The rediscovery of a type specimen of *P.kaduana* at W supersedes the neotypification of Sohmer (1977; ICN, Art. 9.19). Although rather fragmentary, the respective specimen is the only original material known and is here designated as the lectotype of this name (ICN, Art. 9.11). In order to maintain nomenclatural stability, the former neotype *E. Christophersen & E. Hume 1426* (BISH) is additionally designated as an epitype to support the limited lectotype material (ICN, Art. 9.8).

### Psychotria mariniana

Taxon classificationPlantaeGentianalesRubiaceae

(Cham. & Schltdl.) Fosberg, Occas. Pap. Bernice Pauahi Bishop Mus. 23(2): 43, 1962.

Coffea mariniana Cham. & Schltdl., Linnaea 4(1): 35–36, 1829a. ≡ Straussiamariniana (Cham. & Schltdl.) A.Gray, Proc. Amer. Acad. Arts 4: 43, 1860. **Type.** USA. Hawaii: Oahu, Southern Koolau Range, < 730 m alt., 28 Nov to 14 Dec [probably 8–9 Dec] 1816, *L.K.A. von Chamisso s.n.* (lectotype, designated here: W-Endl. 0066414!); Kaeleku, west branch near trail, 1 Jun 1933, *G.W. Russ s.n.* (epitype, designated here: BISH barcode 1010995!, Sohmer 1977: fig. 36! under erroneous collection “*Russ*, 1. July, 1938”).

#### General remarks.

*Psychotriamariniana* (sect. Straussia) is widespread and found on the islands of Kauai, Oahu, Molokai, Lanai and Maui. The species is variable in morphology and habitat preferences and grows in both wet and dry forests (Sohmer 1977, 1978; Wagner et al. 1999). A detailed synonymy and description of the species including lists of specimens, distribution maps, drawings and photos illustrating morphological variations is found in Sohmer (1977: 141–148). According to molecular phylogenetic data, *Psychotriamariniana* belongs to the “*mariniana*” clade comprising also Psychotriahawaiiensis(A.Gray)Fosbergvar.hawaiiensis and *Psychotriawawrae* Sohm. (Nepokroeff et al. 2003).

#### Etymology.

The protologue of *Psychotriamariniana* lacks information about the etymology of the name, but the species appears to be named in honour of the Spanish Don Francisco de Paulo Marín (1774–1837), who is mentioned in Chamisso’s expedition report (1836a: 218, 340ff). Initially an apprentice on a Spanish ship associated with the Malaspina Expedition, he deserted and jumped ship at Nootka Sound (Canada) in 1792. According to Marín’s own account, he was then tricked aboard a ship in San Francisco and kidnapped to Hawaii (Chamisso 1836a: 218, but see Cutter 1980: 20 on the credibility of Marín’s stories). According to archival sources, he joined the U.S. ship *Lady Washington* under the command of Captain John Kendrick and finally reached Oahu in 1793 or 1794 (Gast 1973; Cutter 1980).

Marín settled on the island of Oahu and soon became an influential advisor to the Hawaiian King Kamehameha I, a wealthy merchant, horticulturalist and introducer of many useful plants and animals such as pineapple (Nagata 1985). During both of Chamisso’s visits to Oahu, Marín provided information, advice and logistic support for his collecting activities (Chamisso 1836a: 218, 340ff; Hillebrand 1888: 179).

#### Typification.

The protologue gives the type information as “Legimus in nemorosis montium O-Wahu A. D. 1816” (Chamisso and Schlechtendal 1829a: 35–36). Using the information in the itinerary (Chamisso 1826: 7–8) and diary (Chamisso 1836a: 215–222, 230) allows dating the expedition’s first visit to Oahu from 28 November to 14 December 1816. Details on their collecting activities during that time are found in the diary and point towards higher altitudes of the Koolau Range as the type locality, which is an area where *Psychotriamariniana* frequently occurs today (Sohmer 1977: fig. 37).

Chamisso made his first botanical collections on the island of Oahu on an “old crater behind Honolulu”, which became known as Diamond Head. He subsequently focused his collecting efforts on the forested valleys around Honolulu. Once, he also collected at higher elevations, for which he made an excursion on 8–9 December 1816. He ascended a valley behind Honolulu, crossed the ridge of the Koolau Range and descended towards the coast. The next day, he returned through a much higher mountain pass to the west (Chamisso 1836a: 230). As the only high-elevation area where collections were made during that visit, the type locality “forested mountains” points towards the aforementioned crossing of the Koolau Range. This appears to be supported by a comparison of collecting localities of different species, in which Chamisso indicated lower-elevation sites such as near sea-level habitats, foothills or other special habitat types in a different way (e.g. Chamisso and Schlechtendal 1826a: 167, 1826b: 539, 1827: 36; Chamisso 1830: 44).

**Figure 2.**
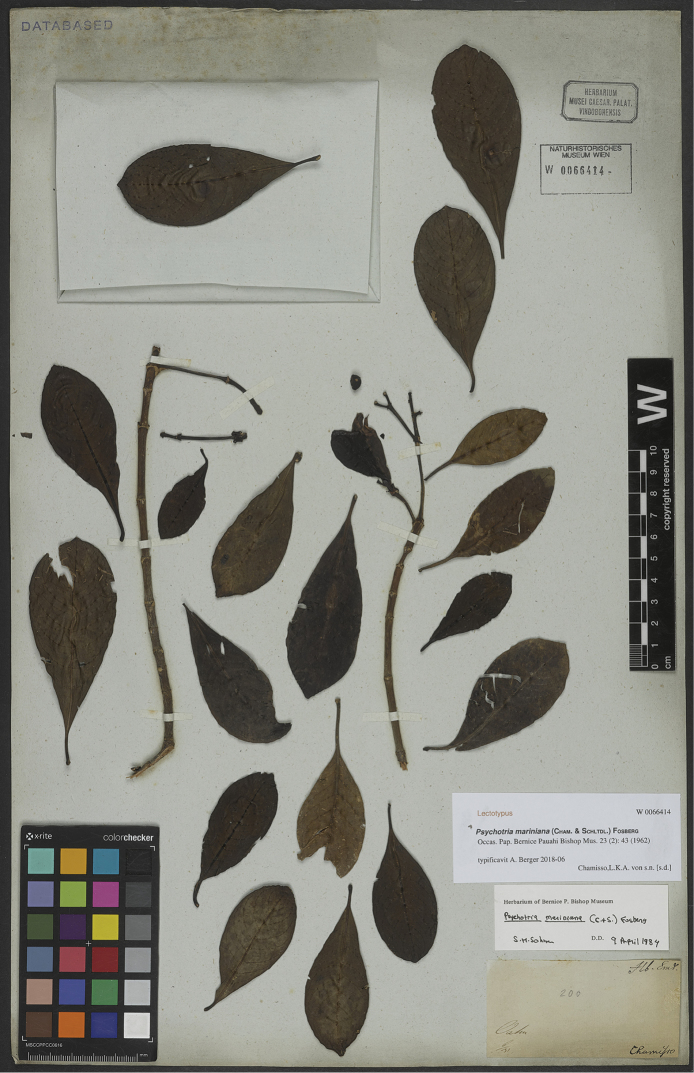
Lectotype of *Psychotriamariniana* (Cham. & Schltdl.) Fosberg collected by L.K.A. von Chamisso during the Romanzoffian Expedition in 1817 (*L.K.A. von Chamisso s.n.*, W-Endl. 0066414). The sheet originates from the private herbarium of S.L. Endlicher, now preserved at the Herbarium of the Natural History Museum, Vienna. Photo: Courtesy of the Natural History Museum, Vienna.

In a similar case as described above, a type specimen of *Psychotriamariniana* is preserved in the private herbarium of Endlicher at W (Figure 2). As with the *P.kaduana* material, the form and details of its label agree with other of Chamisso’s collections, although it lacks any inscriptions in his hand. Instead, it says only “Chamisso”, “Oahu” and “6/31” in ink, as well as “200” in pencil, which was probably added at a later date. The meaning of the numbers is unclear, but the former could refer to the time of acquisition in the herbarium of Endlicher. In 1984, Sohmer confirmed the identification of this specimen as *P.mariniana*.

#### Typification.

As for *Psychotriakaduana*, the rediscovered original material of *P.mariniana* supersedes the neotype designated by Sohmer (1977; ICN, Art. 9.19). This specimen is also incomplete, with two small sterile branchlets and a packet with loose leaves and a single fruit. This specimen is here designated as the lectotype of *P.mariniana* and, in order to maintain nomenclatural stability, the former neotype *G.W. Russ s.n.* (BISH) is here designated as an epitype (ICN, Art. 9.8).

## Supplementary Material

XML Treatment for Psychotria kaduana

XML Treatment for Psychotria mariniana
